# Aberrant Functional Connectivity Between Regions Involved in Belief Evaluation and Processing of Bodily Information in Patients with Somatic Delusions

**DOI:** 10.1192/j.eurpsy.2024.1336

**Published:** 2024-08-27

**Authors:** Y. Panikratova, E. Abdullina, A. Dudina, A. Andrushchenko, G. Kostyuk, D. Romanov, E. Ilina, M. Magomedagaev, P. Iuzbashian, I. Lebedeva

**Affiliations:** ^1^Laboratory of Neuroimaging and Multimodal Analysis, Mental Health Research Center; ^2^ Mental-health Clinic No.1 named after N.A. Alexeev; ^3^Department of Psychiatry and Psychosomatics, I.M. Sechenov First Moscow State Medical University; ^4^Department of borderline mental pathology and psychosomatic disorders, Mental Health Research Center, Moscow, Russian Federation

## Abstract

**Introduction:**

According to the two-factor theory of delusional belief (Coltheart. Ann N Y Acad Sci 2010; 1191 16-26), explaining the presence of a delusion requires a combination of two neuropsychological impairments. The first deficit initially prompts the delusional belief and defines its content, whereas the second deficit – aberrant belief evaluation – interrupts the rejection of a delusional belief and is common for different types of delusions. The second deficit is associated with compromised functioning of the right ventral frontal/anterior insular cortex (r-VF/AI; Darby et al. Brain 2017; 140 497-507). However, neural correlates of the first deficit in different types of delusions remain obscure.

**Objectives:**

The aim of the study was to search for regions whose functional connectivity with r-VF/AI is different between patients with somatic delusions (SD) and persecutory delusions (PD) and to further clarify the results by comparing clinical groups with healthy controls. We hypothesized that each clinical group is characterized by aberrant functional connectivity between a region, associated with poor belief evaluation (r-VF/AI), and a region, presumably associated with a neuropsychological impairment specific to the corresponding type of delusions.

**Methods:**

Patients with delusional disorder or paranoid schizophrenia (*n* = 23) and healthy controls (*n* = 9; 5 females; mean age 36.2 ± 1.3) underwent resting-state fMRI (Philips Ingenia 3T). Nine patients had SD (5 females; mean age 40.3 ± 7.9) and fourteen patients had PD (3 females; mean age 35.6 ± 10.2). The clinical groups were compared in terms of whole-brain functional connectivity of r-VF/AI (ROI-to-voxel analysis in CONN; RRID:SCR_009550; www.nitrc.org/projects/conn). Statistical thresholds were *p* < .005 voxelwise, *p*[FDR] < .05 clusterwise. Each clinical group was compared with controls in terms of functional connectivity between r-VF/AI and previously identified regions with between-group differences in connectivity (ROI-to-ROI analysis). Age was a covariate of no interest in all analyses.

**Results:**

Patients with SD compared to patients with PD and healthy individuals had higher functional connectivity between the r-VF/AI and a cluster in the right precentral and postcentral gyri extending to supramarginal and superior frontal gyri (Figure 1).

**Image:**

**Figure:**
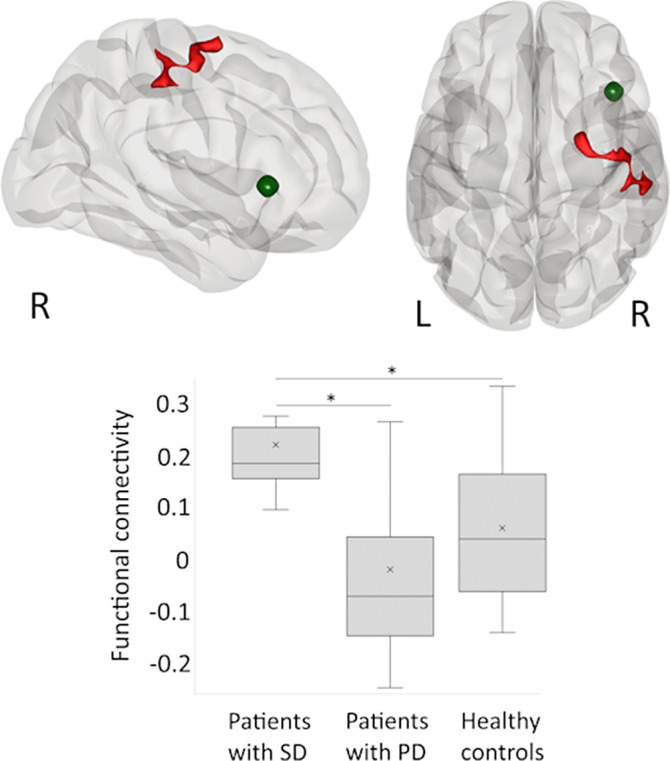

**Conclusions:**

The regions whose functional connectivity with r-VF/AI was aberrant in patients with SD are involved in the processing of tactile, proprioceptive, and visceral information. Our results coincide with a suggestion that the evaluation of beliefs related to bodily sensations is disturbed in patients with SD.

**Research was supported by RFBR grant project 21-515-12007.**

**Disclosure of Interest:**

None Declared

